# Note from Our Oldest Subscriber

**Published:** 1913-07

**Authors:** 


### CORRESPONDENCE.

*This department is intended for the presentation of news and views not coming within the scope of other departments, and particularly to afford opportunity for discussion and criticism of views elsewhere expressed.*

### Note From Our Oldest Subscriber

Three hundred years before Christ, Aristotle and the human race in general declared the human brain is so much stuffing to give shape to the cranium for appearance sake, but within the last century Gall and Spurzheim have launched the science of phrenology on the earth. I am convinced that there is much truth in it and that it cannot be eradicated from the earth. On page 645 of the BUFFALO MEDICAL JOURNAL, Vol. X, can be seen an article of mine on the production of sexes. In the same volume can be seen an article of mine on the constitutions and temperaments. SILAS HUBBARD, M. D., E .Aurora.

